# Portable Home‐Use Triglyceride Meter Demonstrates Good Agreement With Plasma Laboratory Measures During Pregnancies Complicated by Metabolic Disease

**DOI:** 10.1002/edm2.70237

**Published:** 2026-05-09

**Authors:** S. S. Farabi, L. A. Barbour, E. Z. Dunn, C. Ingram, E. Lopez, J. Hinojosa, E. Phillips, N. Hirsch, K. Rolloff, S. Pierce, J. E. Friedman, T. L. Hernandez

**Affiliations:** ^1^ Barnes‐Jewish College Goldfarb School of Nursing Office of Nursing Research St. Louis Missouri USA; ^2^ School of Public Health Washington University in St. Louis St. Louis Missouri USA; ^3^ Division of Nutritional Science & Obesity Medicine Department of Medicine, Washington University in St. Louis School of Medicine St. Louis Missouri USA; ^4^ Department of Medicine, Division of Endocrinology, Metabolism, and Diabetes University of Colorado School of Medicine, Anschutz Medical Campus Aurora Colorado USA; ^5^ Division of Maternal‐Fetal Medicine, Department of Obstetrics and Gynaecology University of Colorado School of Medicine, Anschutz Medical Campus Aurora Colorado USA; ^6^ College of Nursing University of Colorado. Anschutz Medical Campus Aurora Colorado USA; ^7^ Section of Maternal‐Fetal Medicine, Department of Obstetrics and Gynaecology University of Oklahoma Oklahoma City Oklahoma USA; ^8^ Harold Hamm Diabetes Center the University of Oklahoma Health Science Center Oklahoma City Oklahoma USA; ^9^ Children's Hospital Colorado Anschutz Medical Campus Aurora Colorado USA

**Keywords:** agreement, pregnancy, triglyceride meter

## Abstract

**Background:**

Fasting and postprandial triglycerides (PPTG) in obese pregnancies may be stronger predictors of fetal overgrowth than glucose, making them a potential novel treatment target. Measurement of TG currently requires venipuncture in a laboratory, a barrier to collecting repeated measures to understand contributions to fetal growth. Our aim was to evaluate agreement between fingerstick capillary TG using an FDA‐approved point‐of‐care (POC) meter and venipuncture plasma TG (vTG) during fasting and controlled fed conditions within pregnant women.

**Methods:**

Pregnant patients (*n* = 35) with obesity alone (59%) or GDM (41%) (BMI 33 ± 4 kg/m^2^) had fasting vTG and POC TG collected, within ≤ 5 min apart at 25 ± 9 weeks' gestation. A subset (*n* = 23) had PPTG collected at 1‐ and 2‐h after a controlled breakfast test meal (35 ± 2 weeks). Two‐way mixed effects intraclass correlations (ICC) and Bland–Altman plots determined agreement. Paired *t*‐tests were used to compare vTG and POC TG (mean ± SD).

**Results:**

Sixty‐eight paired fasting TG and 52 paired 1‐ and 2‐h PPTG were collected. Fasting vTG were slightly lower than POC TG (181 ± 66 vs. 192 ± 81 mg/dL), as were 1‐h (225 ± 65 vs. 260 ± 76) and 2‐h PPTG (227 ± 70 vs. 249 ± 77; *p* < 0.05 all). The ICC for fasting TG was 0.86 [95% CI: 0.78, 0.91] and 0.84 [95% CI: 0.32, 0.94] for PPTG. The mean % difference (vTG minus POC TG) was −4.6% ± 17.8% for fasting TG and −12.0% ± 12.0% for PPTG.

**Discussion/Conclusion:**

These data suggest good agreement between vTG and POC TG in fasting and PPTG during pregnancies complicated by obesity and GDM. Our findings support the novel approach to utilising a POC TG meter, similar to a glucometer, to conveniently discern the contribution of TG and their potential targets in optimising fetal growth in pregnant patients with obesity and GDM.

## Graphical abstract

Point‐of‐care triglyceride monitors have not been validated in pregnant women. We conducted a study where triglycerides in blood samples were measured using the point‐of‐care meter and a blood sample drawn by a nurse taken at the same time in pregnant women. We took samples at fasting (68 paired samples) and then, in a smaller sample (52 paired samples) at 1‐ and 2‐h after a controlled meal. We found good agreement between the point‐of‐care monitor and lab‐measured triglycerides.

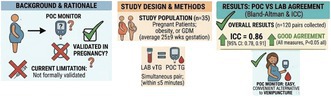

## 1 Introduction

Management of maternal metabolic health in pregnancy is crucial due to its direct impact on maternal and fetal outcomes [1, 2]. It has been shown that triglycerides (TG), both fasting and postprandial (PPTG), are stronger predictors of fetal overgrowth compared to glucose levels, particularly in pregnancies complicated by obesity [3]. Interestingly, pregnant women are often in a postprandial state. We found that the correlation between postprandial TG and neonatal adiposity early in pregnancy was slightly greater (*r* = 0.71, *p* < 0.01) than with fasting TG (*r* = 0.60, *p <* 0.01), and glucose did not add predictive value in regression models [3]. This introduces TG as a potential novel target for therapeutic intervention aimed at optimising fetal development amid metabolic adaptations to pregnancy. However, the conventional method of TG measurement via venipuncture in a laboratory setting is a barrier [4], especially when frequent monitoring or the need to measure postprandial TG is required to fully understand TG fluctuations and their implications on fetal growth. Logistical requirements of laboratory venipuncture, including inconvenient travel to a laboratory and the need for venipuncture, hinder its practical application. Thus, there is a need for more convenient and feasible alternatives, similar to glucometers, for women with gestational diabetes.

Point‐of‐care (POC) home‐use testing, which provides rapid and minimally invasive results through fingerstick capillary blood sampling, offers an alternative to laboratory venipuncture [4]. A POC meter for TG measurements, similar to a glucometer, can enable more frequent monitoring, potentially leading to better management of TG levels and improved maternal and fetal health outcomes. While POC meters have been established to be reliable in non‐pregnant adults [5], the reliability of POC meters has not been widely investigated in pregnancy. Physiologic changes that occur in pregnancy may influence the POC meter reliability for pregnant women. First, most POC devices that use whole blood are sensitive to the haematocrit level. In pregnancy, plasma volume expands significantly more than red blood cell mass, leading to a reduction in haematocrit [6]. This may influence a POC TG meter similar to a glucometer, as a lower haematocrit would mean there is a higher proportion of plasma in the capillary sample, leading to over‐ or under‐estimation of the actual TG concentration [7]. Second, TG levels can increase 2–4 fold during the second and third trimesters under the influence of placental oestrogen and increasing insulin resistance to ensure nutrient availability for the fetus [8]. This is important as high lipid concentrations could change the physical thickness or chemical composition of the blood, which would interfere with the reflectance photometry used by the POC device [9]. Third, pregnant patients with GDM or obesity have higher TGs than pregnant patients with normal weight or in a non‐pregnant population across BMIs; validating the methodology in pregnant women with metabolic disease is clinically important, given this population may be targeted for treatment [10]. Fourth, pregnancy alters peripheral vascular resistance, and peripheral oedema is increased due to increased capillary permeability [11]. This alters the rate of nutrient exchange between the capillaries and the tissues and could create a gap between what is measured in a venous draw (systemic) and what is captured in a fingerstick (capillary bed). Before such devices can be widely adopted in pregnancy, it is essential to establish their accuracy and reliability in comparison to the traditional venipuncture plasma TG (vTG) measurement approach in the pregnant population.

In this study, we aimed to evaluate the agreement between POC TG and vTG measurements within pregnant women during fasting and controlled fed conditions.

## 2 Methods

Samples obtained from pregnant women participating in 3 clinical studies designed to study glucose and lipid metabolism during pregnancy (NCT04349475, NCT02244814, NCT04394806) were used for this study. The first study was a prospective longitudinal cohort (no intervention) in which pregnant women with obesity were enrolled (NCT 04394806). The second was a randomised clinical trial in which women with obesity were assigned to take an omega‐3 fatty acid supplement or a placebo (NCT 04349475). In the third study (NCT02244814), women with diet‐controlled gestational diabetes were randomised to one of two diets just after diagnosis; diets were provided through infant delivery [12]. Samples from baseline were used from the omega‐3 intervention study (NCT 04349475; *n* = 28 paired samples), samples from baseline and follow‐up were used from the longitudinal cohort study (NCT 04394806; *n* = 12 paired samples), and samples from both baseline and post‐intervention were used from the third study (NCT02244814; *n* = 80 paired samples). The studies were approved by the Colorado Multiple Institutional Review Board, and women granted their informed consent for participation. For all 3 studies, women spoke English, had a singleton pregnancy, and were 20–39 y. with a BMI of 26–42 kg/m^2^ at diagnosis. Additionally, for all 3 studies, exclusion criteria included: Fasting TG≥ 400 mg/dL, pre‐existing diabetes, including those with an A1C ≥ 6.5% or random glucose > 200 mg/dL, or a fasting glucose > 105 mg/dL to exclude patients who met criteria for pre‐existing diabetes or at high risk for TG‐related complications or requiring insulin for GDM. Women with GDM met diagnostic criteria by a 100‐g OGTT, and did not require medication at enrollment or during the trial. All samples were collected during either the second (14–28 weeks) or third (28–36 weeks) trimester. BMI did not vary significantly across women between studies (*p* = 0.21, 1‐way ANOVA).

In all 3 studies, women had their blood collected via venipuncture by a registered nurse at the Clinical Research unit at the University of Colorado. Within five minutes of the venipuncture measurement, capillary blood was collected via fingerstick with the POC meter and the assistance of a research assistant. Concurrent blood samples were collected in all three studies after an overnight fast (8–10 h).

In one study (NCT02244814), pregnant women with diet‐controlled gestational diabetes were randomised to either receive a higher complex carbohydrate (CHOICE) or a conventional lower‐carbohydrate diet (LC/CONV) [12]. After fasting, samples were collected (8–10 h), and they had concurrent blood samples collected 1‐ and 2‐h after a standardised breakfast test meal at 30–31 weeks' and then again at 36–37 weeks [12].

Venous plasma TG were analysed from blood collected via venipuncture enzymatically in the laboratory [12, 13] TG were analysed from capillary blood collected via fingerstick (15 uL) using reflectance photometry in the POC meter (CardioCheck PA, PTS Diagnostics) [14].

*Statistical Analysis*: Two‐way mixed effects intraclass correlation coefficients (ICC) and Bland–Altman plots (average of measures on x‐axis and difference between measures on y‐axis) were used to determine agreement [15]. Normality of data was confirmed visually with the Q‐Q plots. Paired two‐tailed *t*‐tests were used to compare fasting, and PPTG laboratory vTG and POC concentrations; a *p* < 0.05 was considered to be statistically significant. For the ICC and Bland–Altman plots, 1‐h and 2‐h PPTG, a sensitivity analysis was conducted, and since average values for the 1‐h and 2‐h were not significantly different between the POC TG (*p* = 0.69) and laboratory vTG values (*p* = 0.93), the groups were combined due to the small numbers for each group. Statistics were completed in STATA (College Station, Texas, USA). Data are reported as mean ± SD.

## 3 Results

A total of 68 paired fasting samples were collected from 35 pregnant women gestational age of 25.9 ± 9 weeks; 15 with obesity alone (BMI 33 ± 4 kg/m^2^) and 20 with gestational diabetes. Fourteen subjects provided two fasting samples. Fifty‐two 1‐ and 2‐h PPTG were collected from 20 women with diet‐controlled gestational diabetes (gestational age 35 ± 2 weeks, BMI 33 ± 4 kg/m^2^). Eight women provided two 1‐h PPTG samples; two women provided two 2‐h PPTG samples Table 1 provides fasting vTG and PPTG measured in the laboratory and via the POC meter. The vTG measurements consistently measured lower than the POC meter TG measurements in both fasting and postprandial conditions: −4.6 ± 17.8 mg/dL for fasting TG, −13.9 ± 13.5 mg/dL for 1‐h PPTG, and −9.4 ± 9.7 mg/dL for 2‐h PPTG. The per cent difference did not significantly differ between the three studies when assessed separately (*p* = 0.51 for 1‐way ANOVA).

**TABLE 1:** Laboratory fasting and postprandial TG values.

	Fasting samples (*n* = 68 paired samples)	1 h Postprandial (*n* = 30 paired samples)	2 h Postprandial (*n* = 22 paired samples)
Lab measured fasting TG, mg/dL	181 ± 66.2	225.4 ± 65.1	226.9 ± 69.7
POC measured fasting TG, mg/dL	192.6 ± 81.0*	260.0 ± 76.0*	249.2 ± 76.8*
% Difference between Lab and POC TG#	−4.6 ± 17.8	−13.9 ± 13.5	−9.4 ± 9.7

*Note:* Data are mean ± SD.

* *p* < 0.05; #: % Difference calculated as (Lab − POC)/Average (Lab &POC) × 100%.

**FIGURE 1:**
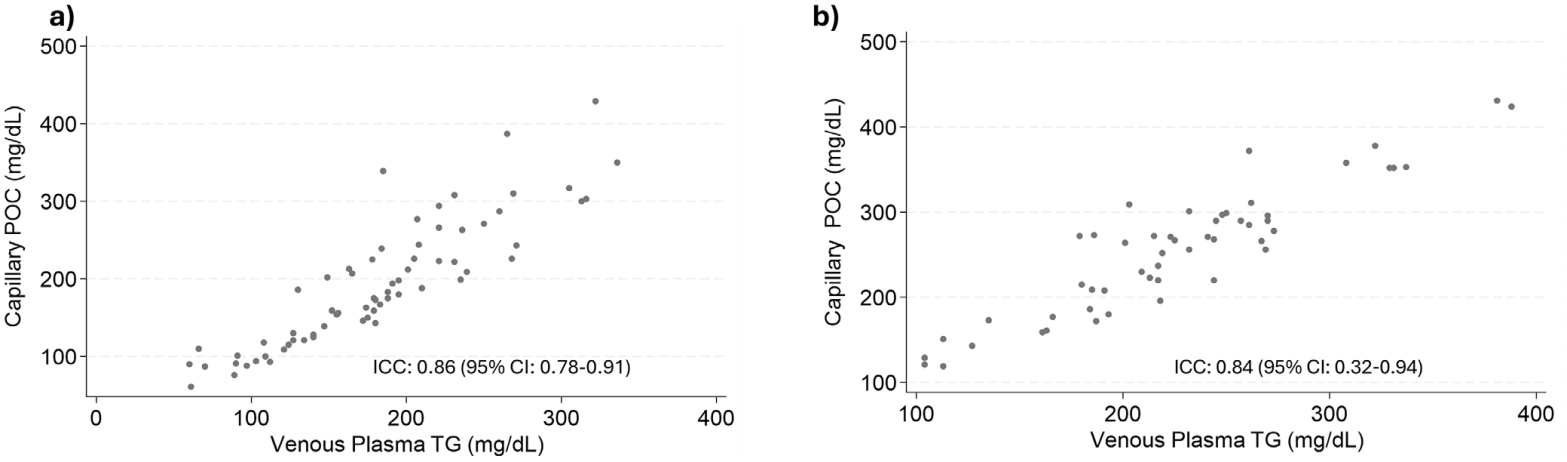
(a) Correlation between Lab and POC measured Fasting TGs; (b) Correlation between Lab and POC measured Postprandial TGs.

**FIGURE 2:**
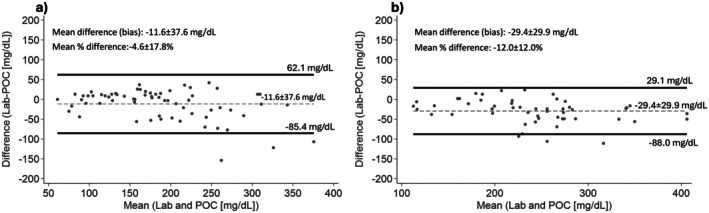
(a) Bland Altman plots show agreement between laboratory and POC fasting TGs Solid black lines indicate 95% limits of agreement (−85.4, 62.1 mg/dL), dashed grey line indicates mean difference (bias) line (−11.6 ± 37.6 mg/dL); (b) Bland Altman plots show agreement between laboratory and POC postprandial TGs Solid black lines indicate 95% limits of agreement (−88.0, 29.1), dashed grey line indicates mean difference (bias) line (−29.4 ± 29.9 mg/dL).

The ICC for vTG vs. POC fasting TG was 0.86 [95% CI: 0.78, 0.91] (Figure 1a), and 0.84 [95% CI: 0.32, 0.94] for PPTG (Figure 1b). Further, Bland Altman plots revealed agreement between the laboratory and POC measures in fasting and 1–2 h postprandial conditions. The average mean per cent difference between vTG and POC TG was −4.6% ± 17.8% for fasting concentrations (Figure 2a) and −12.0% ± 12.0% for postprandial concentrations (Figure 2b).

## 4 Discussion

The findings of this analysis across our studies of triglyceride metabolism during pregnancy suggest that there is a good agreement between conventional vTG and POC TG measurements, both in fasting and postprandial states, for women with overweight/obesity or GDM. The ICCs for fasting TG and PPTG were 0.86 and 0.84, respectively, indicating consistency between the two methods. Although the POC TG readings were slightly higher than the vTG measurements, with differences of +11 mg/dL, +35 mg/dL, and +22 mg/dL for fasting, 1‐h, and 2‐h PPTG, respectively, these variances fell within a range that is clinically acceptable for glucometers [16] and the findings are consistent with studies in non‐pregnant populations [5].

In this study, we found that the POC TG readings were slightly but consistently higher than the vTG measurements. This may be due to the fact that POC devices use whole blood derived from a capillary bed, while the vTG was measured from centrifuged plasma. This may be due to the fact that in pregnancy, haematocrit is lower due to an expansion of plasma volume [6]. Since POC devices use algorithms for whole blood, which account for haematocrit levels, a lower haematocrit may lead to an over‐estimation by the POC device than the actual TG concentration [7]. Capillary permeability is increased in pregnancy due to peripheral vascular resistance [11]. The increased permeability may alter the rate of nutrient exchange between the capillaries and the tissues, resulting in a slightly higher level of TG presented at the capillary bed (POC device) than what is measured in the plasma (vTG). Future studies may be needed to determine if algorithms for POC devices should be updated for more accurate use in pregnant women with obesity and GDM.

In this analysis, we found a 4.6% difference between vTG and POC meter fasting TG, and a 12.0% difference for postprandial TG. These differences fall within FDA and International Organization of Standardization acceptable ranges for POC glucometers (< 15%) used for glucose measurement [16]. Although the differences between vTG and POC measures are less than the 15% difference used for POC glucometers, there are no current standards for POC TG meters, and glucose and TG monitors are analytically and clinically distinct measurement approaches. Future research and guidance are necessary to establish agreement ranges for POC TG meters in research and clinical care of pregnant women. While the ICC value for the postprandial TGs of 0.84 indicates good agreement, the 95% confidence interval was wide (0.32–0.94), indicating instability of the estimate. This is likely due to a small sample size of women who provided paired postprandial samples. The larger differences for postprandial TG may reflect the dynamic nature of TG concentrations after meals. Fasting TG reflect steady‐state VLDL‐TG from the liver, while postprandial TG reflects gut‐derived chylomicron‐TG, peaking 2–3 h after a meal [17].

The contributions to fetal overgrowth, and more clinically important, excess fetal fat accretion, remain an elusive and highly significant clinical challenge in pregnancies affected by obesity and gestational diabetes. Even with treatment of gestational diabetes, rates of large‐for‐gestational‐age infants (macrosomia) remain higher than the background prevalence [18]. In the Hyperglycemia and Adverse Pregnancy Outcomes Study, most macrosomia occurred in women with obesity outside of gestational diabetes [19], and the majority of LGA infants are born to mothers with obesity, not diabetes, suggesting a mechanism beyond glucose control. With mounting evidence pointing to TG as a driver of fetal overgrowth [20], especially in pregnancies complicated with obesity, the ability to use a POC TG is a promising tool for the future that could aid in targeting the optimal monitoring and management of TG in pregnancies complicated by obesity. Future studies are needed to confirm and extend our findings, as well as to link the use of a POC TG monitor to clinical findings that include fetal growth and newborn body composition. Regular and convenient TG monitoring at home could enable more personalised and timely interventions, potentially reducing the risk of fetal overgrowth, just as glucometers revolutionised the management of diabetes in pregnancy [3].

Strengths of this study include the collection of venous and POC TG measures within ≤ 5 min after a consistent fasting period. Moreover, PPTG measures were collected within ≤ 5 min of each other during a controlled breakfast test meal with a known macronutrient composition. Both samples were collected by trained research personnel. The POC TG device requires a larger blood sample (15 uL) and use of a capillary tube for application to the test strip compared to a glucometer (5 uL), where blood is directly applied to the test strip; however, this has been quite easily accomplished by the majority of mothers in our ongoing studies. In our current studies, women use both a glucometer and the POC TG meter at home and use one large blood drop to test both POC glucose and TG. Good acceptability and ease of use are reported.

### 4.1 Limitations

Our study is limited in that our findings may not be generalisable to women with normal weight in pregnancy, although they may be likely to have similar TG ranges as in the non‐pregnant population across BMIs, previously validated. Further validation in these populations is needed. Further, since this is a secondary analysis of clinical trials with pooled data, our exclusion criteria for the studies may limit the applicability of the findings to broader clinical populations (e.g., individuals with severe hypertriglyceridemia, preexisting diabetes, or less well‐controlled glucose levels). While the ICC values indicate good agreement, the confidence intervals for the ICCs were wide, potentially due to our small, heterogeneous sample. Future research should continue to explore this promising avenue, incorporating larger sample sizes and diverse populations to validate these findings further and establish standardised protocols for POC TG monitoring in pregnancy.

## 5 Conclusions

In conclusion, our novel data support that this POC TG meter is reliable for monitoring TG concentrations in pregnant women with obesity or GDM, offering a convenient approach to monitoring and potentially improving metabolic health for both the mother and her baby. This method holds potential not only for enhancing clinical outcomes, such as normalising fetal growth, but also for empowering patients through more accessible and less invasive testing options.

## Author Contributions

**S. S. Farabi:** conceptualization, writing – original draft, investigation, methodology, formal analysis. **S. Pierce:** writing – review and editing, supervision, data curation. **K. Rolloff:** writing – review and editing, methodology, data curation, project administration. **C. Ingram:** project administration, writing – review and editing, data curation. **J. Hinojosa:** data curation, writing – review and editing. **L. A. Barbour:** conceptualization, funding acquisition, writing – review and editing, supervision. **J. E. Friedman:** supervision, funding acquisition, writing – review and editing, resources. **N. Hirsch:** funding acquisition, writing – review and editing, data curation, project administration. **E. Z. Dunn:** data curation, writing – review and editing, project administration. **E. Phillips:** data curation, writing – review and editing. **T. L. Hernandez:** resources, project administration, investigation, conceptualization, writing – review and editing, supervision, funding acquisition. **E. Lopez:** writing – review and editing, data curation.

## Funding

This work was supported by the National Institute of Diabetes and Digestive and Kidney Diseases (R01DK101659, T32DK007446), the National Center for Advancing Translational Sciences (UL1 TR002535), and Janssen Research and Development.

## Ethics Statement

All study procedures were reviewed and approved by the University of Colorado Anschutz Medical Center Institutional Review Board.

## Consent

Written informed consent was obtained from all study participants.

## Conflicts of Interest

The authors declare no conflicts of interest.

## Data Availability

The data that support the findings of this study are available on request from the corresponding author. The data are not publicly available due to privacy or ethical restrictions.
